# Deep Learning-Based Body Composition Analysis Predicts Outcome in Melanoma Patients Treated with Immune Checkpoint Inhibitors

**DOI:** 10.3390/diagnostics11122314

**Published:** 2021-12-09

**Authors:** Anton Faron, Nikola S. Opheys, Sebastian Nowak, Alois M. Sprinkart, Alexander Isaak, Maike Theis, Narine Mesropyan, Christoph Endler, Judith Sirokay, Claus C. Pieper, Daniel Kuetting, Ulrike Attenberger, Jennifer Landsberg, Julian A. Luetkens

**Affiliations:** 1 Department of Diagnostics and Interventional Radiology, Venusberg Campus 1, University Hospital Bonn, 53127 Bonn, Germany; Anton.Faron@gmx.de (A.F.); nikolaopheys@hotmail.de (N.S.O.); sebastian.nowak@ukbonn.de (S.N.); sprinkart@uni-bonn.de (A.M.S.); alexander.isaak@ukbonn.de (A.I.); maike.theis@ukbonn.de (M.T.); narine.mesropyan@ukbonn.de (N.M.); christoph.endler@ukbonn.de (C.E.); claus_christian.pieper@ukbonn.de (C.C.P.); daniel.kuetting@ukbonn.de (D.K.); ulrike.attenberger@ukbonn.de (U.A.); 2 Quantitative Imaging Lab Bonn (QLaB), Venusberg Campus 1, University Hospital Bonn, 53127 Bonn, Germany; 3 Center of Integrated Oncology (CIO) Bonn, Department of Dermatology and Allergy, Venusberg Campus 1, University Hospital Bonn, 53127 Bonn, Germany; judith.sirokay@ukbonn.de (J.S.); jennifer.landsberg@ukbonn.de (J.L.)

**Keywords:** oncologic imaging, CT, imaging biomarkers, sarcopenia, artificial intelligence

## Abstract

Previous studies suggest an impact of body composition on outcome in melanoma patients. We aimed to determine the prognostic value of CT-based body composition assessment in patients receiving immune checkpoint inhibitor therapy for treatment of metastatic disease using a deep learning approach. One hundred seven patients with staging CT examinations prior to initiation of checkpoint inhibition between January 2013 and August 2019 were retrospectively evaluated. Using an automated deep learning-based body composition analysis pipeline, parameters for estimation of skeletal muscle mass (skeletal muscle index, SMI) and adipose tissue compartments (visceral adipose tissue index, VAI; subcutaneous adipose tissue index, SAI) were derived from staging CT. The cohort was binarized according to gender-specific median cut-off values. Patients below the median were defined as having low SMI, VAI, or SAI, respectively. The impact on outcome was assessed using the Kaplan–Meier method with log-rank tests. A multivariable logistic regression model was built to test the impact of body composition parameters on 3-year mortality. Patients with low SMI displayed significantly increased 1-year (25% versus 9%, *p* = 0.035), 2-year (32% versus 13%, *p* = 0.017), and 3-year mortality (38% versus 19%, *p* = 0.016). No significant differences with regard to adipose tissue compartments were observed (3-year mortality: VAI, *p* = 0.448; SAI, *p* = 0.731). On multivariable analysis, low SMI (hazard ratio (HR), 2.245; 95% confidence interval (CI), 1.005–5.017; *p* = 0.049), neutrophil-to-lymphocyte ratio (HR, 1.170; 95% CI, 1.076–1.273; *p* < 0.001), and Karnofsky index (HR, 0.965; 95% CI, 0.945–0.985; *p* = 0.001) remained as significant predictors of 3-year mortality. Lowered skeletal muscle index as an indicator of sarcopenia was associated with worse outcome in patients with metastatic melanoma receiving immune checkpoint inhibitor therapy.

## 1. Introduction

The approval of ipilimumab as the first immune checkpoint inhibitor for treatment of advanced melanoma by the United States Food and Drug Administration in 2011 introduced a new era in cancer treatment [1,2,3]. Ipilimumab is a monoclonal antibody targeting the cytotoxic T-lymphocyte associated protein 4 (CTLA-4) receptor, leading to T-cell activation. Two other immune checkpoint inhibitors (nivolumab and pembrolizumab) were introduced shortly thereafter for treatment of advanced melanoma, targeting the programed cell death 1 (PD1) signaling pathway [4,5,6]. These three immune checkpoint inhibitors were proven to be highly effective for treatment of advanced melanoma, and therefore, currently are considered as part of the standard of care [7]. However, response rates were observed to vary and since severe treatment-related toxicities may occur, identification of predictive factors has evolved as a field of active investigation [8]. 

Previous studies showed that aspects of body constitution may have an impact on outcome in various malignancies, including melanoma patients [9,10,11,12,13]. In a recent report, a survival benefit was demonstrated for overweight patients receiving immune checkpoint inhibitors for treatment of metastatic melanoma, and it was hypothesized that this observation may be related to a higher amount of skeletal muscle mass [14]. As with many other malignant diseases, computed tomography (CT) is typically performed for staging purposes prior to treatment initiation in patients with advanced melanoma. Thereby, beyond the primary diagnostic purpose, body constitution may be evaluated from these CT scans. In other patient cohorts with oncologic and cardiovascular diseases, this so-called “opportunistic imaging” approach was shown to reveal a promising prognostic about survival duration and clinical outcome [9,15]. Recently, it was demonstrated that deep learning algorithms may be used to obtain body composition parameters from CT examinations in an automated fashion [16]. Such an approach may be of great clinical interest, as automatization may facilitate clinical applicability.

With this study, we aimed to determine the feasibility and clinical potential of automated body composition analysis from staging CT examinations using a deep learning pipeline to predict outcome in melanoma patients receiving immune checkpoint inhibitor therapy.

## 2. Materials and Methods

### 2.1. Study Population

The local ethics committee approved this retrospective study with a waiver of need for written informed consent. Patients who started immune checkpoint inhibitor therapy for treatment of metastatic melanoma between January 2013 and August 2019 at our tertiary academic center were retrospectively evaluated. Among these, patients with available and complete thoracoabdominal staging CT scans immediately prior to initiation of first-time treatment with immune checkpoint inhibitors were identified. Medical records of the included patients were reviewed to retrieve baseline clinical and laboratory variables. Disease stage was classified according to the eighth edition of the American Joint Committee on Cancer (AJCC) melanoma staging system. Patients with prior exposure to immune checkpoint inhibitor therapy were excluded from the analysis.

### 2.2. Image Analysis

**Figure 1:**
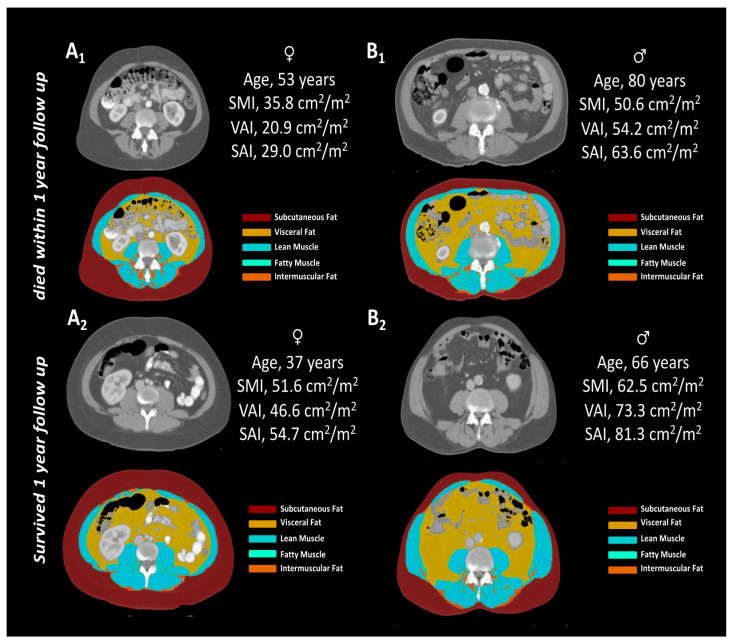
Exemplary patients from the study population. Female (**A1**,**A2**) and male (**B1**,**B2**) patients who passed away (**A1**,**B1**) or were alive (**A2**,**B2**) 1 year after initial staging CT for treatment initiation of immune checkpoint inhibitor therapy alongside obtained body composition metrics (SMI, VAI, SAI). Abbreviations: SMI, skeletal muscle index; VAI, visceral adipose tissue index; SAI, subcutaneous adipose tissue index.

Transverse cross-sections at the level of the intervertebral disc space between the third and fourth lumbar vertebra (L3/4) were used for body composition analysis, as connective tissue areas (skeletal muscle area, subcutaneous fat area, visceral fat area) at this landmark were previously shown to correlate well with compartment volumes [17]. Single-slice images at the L3/4 level were exported for each patient from the local picture archiving and communication system (IMPAX EE, Dedalus HealthCare) to a dedicated workstation. A deep learning model for automated body composition analysis was used for tissue segmentation [16]. Segmented images were visually inspected by a radiologist with 3 years of experience in body composition analysis and adjusted manually, if necessary. The radiologist was blinded to patient data and unaware of patient outcomes. Exemplary images are provided in Figure 1. 

### 2.3. Statistical Analysis

To account for differences in patient constitution, derived connective tissue compartment areas of skeletal muscles, subcutaneous fat, and visceral fat were normalized for patient height using the formula:

$$connective\;tissue\;compartment\;index=\frac{connective\;tissue\;compartment\;area\;\left({\mathrm{cm}}^{2}\right)}{{\left(patient\;body\;height\;\left(m\right)\right)}^{2}}$$

and the respective connective tissue compartment indices (skeletal muscle index, SMI; visceral adipose tissue index, VAI; subcutaneous adipose tissue index, SAI) were calculated. Patients were binarized according to median SMI, SAI, and VAI using gender-specific cut-offs. Patients with values below the gender-specific median were defined to have low SMI, low SAI, and low VAI, respectively. The Shapiro–Wilk test was used to check continuous data for normal distribution. If normally distributed, continuous data are provided as means with standard deviation and otherwise expressed as medians with 25th and 75th interquartile range (IQR). Differences between groups were assessed using unpaired t-test and Mann–Whitney U test for parametric and nonparametric testing, respectively. Categorical data are expressed as total numbers and frequencies. The χ^2^ test was used to compare categorical data between the groups. Kaplan–Meier curves with log-rank tests were calculated to compare survival of patients with high and low values of the respective connective tissue compartment indices (SMI, SAI, VAI) for a follow-up of up to 3 years. A multivariate logistic regression model with stepwise forward selection including factors that were significantly associated with 3-year mortality on univariate analysis was built, and results were displayed as hazard ratios (HR) with 95% confidence intervals (CI). Statistical analysis was performed using SPSS Statistics 25 (IBM, Armonk, NY, USA) and Prism 8 (GraphPad software, La Jolla, CA, USA). A level of *p* < 0.05 was considered to indicate a significant difference. 

## 3. Results

### 3.1. General Patient Characteristics

Of 114 eligible patients, seven were excluded due to incomplete baseline anthropometric characteristics (body height, body weight) at time of staging CT. Accordingly, a total of 107 patients (70 male; mean age, 62 ± 15 years) were included. A total of 30 patients (28%) were classified as stage III according to AJCC, while the remainder were classified as stage IV (N = 77, 72%). The majority of patients were treated with PD-1 monotherapy (nivolumab or pembrolizumab, 70/107, 65%), while the remainder received either a combination therapy of PD-1 and CTLA-4 (nivolumab plus ipilimumab, 20/107, 19%) or CTLA-4 monotherapy (ipilimumab, 17/107, 16%). Included patients had a mean body mass index (BMI) of 27 ± 5 kg/m^2^ with a mean body height of 1.74 ± 0.09 m and a mean body weight of 82 ± 18 kg. Male and female patients did not differ significantly with regard to median age and BMI (*p* > 0.05; Table 1). However, male patients were significantly taller (1.79 m; interquartile range (IQR), 1.72–1.83 versus 1.64 m; IQR 1.62–1.68; *p* < 0.001) and had a significantly higher median body weight (85 kg; IQR 75–97 versus 68 kg; IQR 59–82; *p* < 0.001) compared to female patients. On body composition analysis, male patients displayed significantly increased median SMI (51.9 cm^2^/m^2^; IQR 46.7–56.9 versus 41.0 cm^2^/m^2^; IQR 36.8–44.5; *p* < 0.001), VAI (69.3 cm^2^/m^2^; IQR 47.2–91.6 versus 30.7 cm^2^/m^2^; IQR 13.4–57.0; *p* < 0.001), and SAI (77.4 cm^2^/m^2^; IQR 54.9–99.1 versus 38.1 cm^2^/m^2^; IQR 19.0–64.2; *p* < 0.001) compared to female patients. 

### 3.2. Impact of Body Composition on Outcome

**Figure 2:**
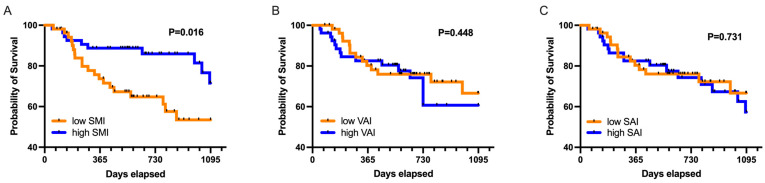
Kaplan–Meier curves illustrating 3-year mortality of patients with high compared to low (**A**) skeletal muscle index (SMI), (**B**) visceral adipose tissue index (VAI), and (**C**) subcutaneous adipose tissue index (SAI).

A total of 30 patients (17 male) died within a follow-up period of 3 years. Those patients did not differ significantly with regard to mean VAI (53.0 ± 31.7 cm^2^/m^2^ versus 61.9 ± 36.8 cm^2^/m^2^, *p* = 0.248) and SAI (60.8 ± 33.1 cm^2^/m^2^ versus 70.0 ± 38.4 cm^2^/m^2^, *p* = 0.251) compared to survivors. However, these patients tended to display a decreased mean SMI (46.1 ± 7.7 cm^2^/m^2^ versus 49.6 ± 9.1 cm^2^/m^2^, *p* = 0.066) compared to survivors. As distinct differences in body composition patterns between male and female patients were observed, the cohort was binarized according to median SMI, VAI, and SAI based on gender-specific cut-offs (Table 1). Patients with values above the gender-specific median were defined to have high SMI, VAI, or SAI, while patients with values below the respective median were termed to have low SMI, VAI, or SAI. On Kaplan–Meier analysis, patients with low SMI displayed significantly increased 1-year (25% versus 9%, *p* = 0.035), 2-year (32% versus 13%, *p* = 0.017), and 3-year mortality (38% versus 19%, *p* = 0.016, Figure 2). Patients with low SMI were older (71 years; IQR 57–79 versus 59 years; IQR 47–69; *p* = 0.001), had higher BMI (25 kg/m^2^; IQR 23–28 versus 28 kg/m^2^; IQR 25–31; *p* = 0.002), and showed increased levels of lactic acid dehydrogenase (LDH, 214 U/L; IQR 186–301 versus 197 U/L; IQR 174–245; *p* = 0.020) at time of treatment initiation compared to patients with high SMI (Table 2). No significant differences in 3-year mortality regarding adipose tissue compartments were observed (low versus high VAI, 26% versus 30%, *p* = 0.48; low versus high SAI, 26% versus 30%, *p* = 0.731). On multivariable risk factor analysis, low SMI (hazard ratio (HR), 2.245; 95% confidence interval (CI), 1.005–5.017; *p* = 0.049), neutrophil-to-lymphocyte ratio (HR, 1.170; 95% CI, 1.076–1.273; *p* < 0.001), and Karnofsky index (HR, 0.965; 95% CI, 0.945–0.985; *p* = 0.001) remained as significant predictors of 3-year mortality (Table 3).

## 4. Discussion

In this study, the feasibility and clinical potential of automated body composition assessment from staging CT was evaluated in melanoma patients receiving immune checkpoint inhibitor therapy. For this purpose, a recently published automated deep learning pipeline was applied [16]. Patients with lowered skeletal muscle index displayed increased mortality rates up to 3 years after treatment initiation, while amounts of adipose tissue compartments were not observed to affect mortality. 

Several studies illustrated an impact of body composition on outcome in various malignancies [9,10,11,13]. For instance, in another report including patients treated with ipilimumab for metastatic melanoma, sarcopenia was related to lowered survival, and increased skeletal muscle fat infiltration—indicating lowered muscle quality—was found to be associated with a higher likelihood of immune-related adverse events [13]. The outstanding role of cross-sectional imaging to determine body composition was stressed in these studies, as sarcopenia may be masked by obesity and thus may remain unrecognized in a clinical setting. Cross-sectional imaging has the potential to overcome this issue, as it allows for quantitative evaluation of tissue compartments irrespective of outward body constitution [17]. 

It is well known that the skeletal muscle system fulfills multifarious functions for the integrity of the human body, which go far beyond simple locomotion. For instance, the skeletal muscle system produces glutamine, which is a main energy resource for rapidly dividing cells, such as leukocytes [18]. Moreover, skeletal muscle tissue also secretes myokines, which, among several other processes, also seem to modulate the immune system [19]. Therefore, the integrity of the skeletal muscle system may be considered particularly relevant regarding therapies affecting the immune systems, such as immune checkpoint inhibitor therapy. 

In our cohort, both male and female patients with lower gender-specific amounts of skeletal muscles had higher mortality rates, up to 3 years after initiation of immune checkpoint inhibitor therapy. These patients were older but did not differ with regard to overall functional performance, as indicated by Karnofsky index. Unlike these routinely available clinical factors, low SMI was related to higher mortality rates on multivariate risk factor analysis in our cohort. This observation may indicate that in melanoma patients treated with immune checkpoint inhibitors, assessment of skeletal muscle mass may reveal additional prognostic information that is not captured by routinely available clinical factors. From a clinical perspective, this observation may be considered particularly interesting, as information on body composition is readily available from staging CT, which again is routinely performed prior to treatment initiation in these patients. Moreover, automated extraction using deep learning algorithms, as the one used in our study [16], may further facilitate its clinical use. 

Interestingly, we observed a significant difference in BMI values between patients with high and low skeletal muscle mass in our cohort. In fact, patients with increased skeletal muscle amount (high SMI group) had a median BMI value of 28 kg/m^2^ and demonstrated increased survival rates compared to patients from the low SMI group with a median BMI of 25 kg/m^2^. This finding is in line with a phenomenon termed “obesity paradox”, which was recently observed in melanoma patients treated with immune checkpoint inhibitors [14]. In this previous report, a survival advantage was observed for overweight and class I obese patients compared to patients with normal BMI values, as well as those with class II and III obesity. The survival advantage was described to be mainly driven by male patients with higher serum creatinine levels. As the serum creatinine level is considered as a laboratory indicator of skeletal muscle mass, it was hypothesized that the observed survival advantage was caused by a higher muscle mass of these patients [14]. Our findings confirmed and extended these previous insights, as we observed a survival advantage for males and females with higher amounts of skeletal muscle mass, which again also had higher BMI values compared to patients with lowered skeletal muscle mass, respectively. 

Regarding laboratory markers, patients with lower SMI showed increased baseline LDH values in our cohort. Moreover, we observed neutrophil-to-lymphocyte ratio to be significantly associated with survival. Although this was not the primary focus of our study, these observations may substantiate insights from previous literature, indicating a prognostic potential of distinct baseline laboratory markers, such as LDH or neutrophile count, in melanoma patients receiving immune checkpoint inhibitor treatment [20,21,22]. Another interesting observation of our study was that although patients with low and high SMI values did not differ regarding disease severity as indicated by AJCC stages, we observed that patients with high SMI values more frequently received combination immune checkpoint inhibitor therapy. Since patients with high SMI values were also younger in our cohort, possibly this observation may be explained by the fact that in advanced melanoma, it was recently recommended to use combination immune checkpoint inhibitor therapy preferably in younger patients, while immune checkpoint inhibitor monotherapy should be applied in elderly subjects [23]. This issue may be further addressed in future studies.

We acknowledge several limitations of our study. First, as with other retrospective single-center studies, results may not be directly transferable to other patient cohorts and, therefore, conclusions must be drawn with caution. However, as indicated above, results of our exploratory analysis were in line with previous literature, suggesting a high prognostic relevance of easy obtainable measurements, which are readily available from routinely performed clinical CT examinations and, therefore, may be easily integrated into routine clinical work-up. We are aware of the comparatively small number of patients within this study, which is primary related to the single-center setting. Taken together, our results warrant larger and particularly prospective studies, which may help to further validate its results. In this regard, an automated deep learning approach, such as the one used in our study, may be considered particularly attractive, as it allows for time-efficient and objective extraction of the prognostic information and also may ease comparability in a multi-center setting. Moreover, these studies may also address a potential impact of immune-related adverse events, which were not available for analysis in our study.

To conclude, this study investigated the prognostic value of body composition assessment in melanoma patients receiving immune checkpoint inhibitor therapy. Patients with lowered skeletal muscle mass displayed increased mortality rates, up to 3 years after treatment initiation. As lowered skeletal muscle mass was identified as an independent predictor of mortality, our results indicated that body composition assessment may reveal additional prognostic information for risk stratification in these patients and that with the help of deep learning, it is feasible to extract the relevant information from routine clinical CT examinations in an automated fashion, which may further improve clinical applicability. 

## Figures and Tables

**Table 1:** Baseline anthropometric characteristics of the study population. Values are provided as median with interquartile range. Mann–Whitney U test was used to compare values between male and female patients.

Variable	Male (N = 70)	Female (N = 37)	*p* Value
Age (y)	67 (55–76)	59 (49–76)	0.325
Body Height (m)	1.79 (1.72–1.83)	1.64 (1.62–1.68)	<0.001
Body Weight (kg)	85 (75–97)	68 (59–82)	<0.001
Body Mass Index (kg/m^2^)	27 (24–30)	25 (23–31)	0.304
Skeletal Muscle Index (cm^2^/m^2^)	51.9 (46.7–56.9)	41.0 (36.8–44.5)	<0.001
Visceral Adipose Tissue Index (cm^2^/m^2^)	69.3 (47.2–91.6)	30.7 (13.4–57.0)	<0.001
Subcutaneous Adipose Tissue Index (cm^2^/m^2^)	77.4 (54.9–99.1)	38.1 (19.0–64.2)	<0.001

**Table 2:** Clinical characteristics of patients with high and low skeletal muscle index (SMI). Abbreviations: PD-1, programmed cell death 1 inhibitor (nivolumab or pembrolizumab), CTLA-4, cytotoxic T-lymphocyte-associated protein 4 inhibitor (ipilimumab), AJCC, American Joint Committee on Cancer. Continuous data are provided as median with interquartile ranges, while categorical data are expressed as total numbers and frequencies. Mann–Whitney U test and χ^2^ test were used for group comparison, as applicable.

Variable	Low SMI (N = 53)	High SMI (N = 54)	*p* Value
Age (years)	71 (57–79)	59 (47–69)	0.001
Body Mass Index (kg/m^2^)	25 (23–28)	28 (25–31)	0.002
Karnofsky Index	100 (90–100)	100 (100–100)	0.070
Lactic Acid Dehydrogenase (U/l)	214 (186–301)	197 (174–245)	0.020
Neutrophile-to-Lymphocyte Ratio	2.8 (1.9–4.3)	2.6 (2.0–3.9)	0.971
PD-1 Monotherapy	37 (70%)	33 (61%)	0.418
CTLA-4 Monotherapy	10 (19%)	7 (13%)	0.439
PD-1 + CTLA Combination Therapy	6 (11%)	14 (26%)	0.081
AJCC stage IV	38 (72%)	39 (72%)	0.952

**Table 3:** Predictors of 3-year mortality in melanoma patients receiving immune checkpoint inhibitor therapy. Predictors were determined using Cox regression analysis. Variables that were significantly associated with 3-year mortality on univariate analysis were entered to the multivariable model using stepwise forward selection. Hazard ratios are provided with 95% confidence interval. Abbreviations: NLR, neutrophil-to-lymphocyte ratio; SMI, skeletal muscle index; BMI, body mass index; LDH, lactic acid dehydrogenase.

Variable	Univariate Analysis		Multivariate Analysis	
	Hazard Ratio (95% Confidence Interval)	*p* Value	Hazard Ratio (95% Confidence Interval)	*p* Value
Sex	0.561 (0.272–1.157)	0.118	-	-
Age	1.010 (0.985–1.035)	0.428	-	-
BMI	0.977 (0.900–1.060)	0.570	-	-
Low SMI	2.464 (1.151–5.278)	0.020	2.245 (1.005–5.017)	0.049
Karnofsky Index	0.963 (0.944–0.982)	<0.001	0.965 (0.945–0.985)	0.001
NLR	1.158 (1.066–1.259)	0.001	1.170 (1.076–1.273)	<0.001
LDH	1.000 (1.000–1.000)	0.675	-	-

## Data Availability

The data presented in this study are available on reasonable request from the corresponding author. The data are not publicly available due to privacy restrictions.
